# Chronic Anatabine Treatment Reduces Alzheimer’s Disease (AD)-Like Pathology and Improves Socio-Behavioral Deficits in a Transgenic Mouse Model of AD

**DOI:** 10.1371/journal.pone.0128224

**Published:** 2015-05-26

**Authors:** Megha Verma, David Beaulieu-Abdelahad, Ghania Ait-Ghezala, Rena Li, Fiona Crawford, Michael Mullan, Daniel Paris

**Affiliations:** 1 The Roskamp Institute, Sarasota, Florida, 34243, United States of America; 2 The Open University, Walton Hall, Milton Keynes, Buckinghamshire, MK7 6AA, United Kingdom; 3 Rock Creek Pharmaceuticals, Sarasota, Florida, 34243, United States of America; University of Lancaster, UNITED KINGDOM

## Abstract

Anatabine is a minor tobacco alkaloid, which is also found in plants of the Solanaceae family and displays a chemical structure similarity with nicotine. We have shown previously that anatabine displays some anti-inflammatory properties and reduces microgliosis and tau phosphorylation in a pure mouse model of tauopathy. We therefore investigated the effects of a chronic oral treatment with anatabine in a transgenic mouse model (Tg PS1/APPswe) of Alzheimer’s disease (AD) which displays pathological Aβ deposits, neuroinflammation and behavioral deficits. In the elevated plus maze, Tg PS1/APPswe mice exhibited hyperactivity and disinhibition compared to wild-type mice. Six and a half months of chronic oral anatabine treatment, suppressed hyperactivity and disinhibition in Tg PS1/APPswe mice compared to Tg PS1/APPswe receiving regular drinking water. Tg PS1/APPswe mice also elicited profound social interaction and social memory deficits, which were both alleviated by the anatabine treatment. We found that anatabine reduces the activation of STAT3 and NFκB in the vicinity of Aβ deposits in Tg PS1/APPswe mice resulting in a reduction of the expression of some of their target genes including *Bace1*, *iNOS* and *Cox-2*. In addition, a significant reduction in microgliosis and pathological deposition of Aβ was observed in the brain of Tg PS1/APPswe mice treated with anatabine. This is the first study to investigate the impact of chronic anatabine treatment on AD-like pathology and behavior in a transgenic mouse model of AD. Overall, our data show that anatabine reduces β-amyloidosis, neuroinflammation and alleviates some behavioral deficits in Tg PS1/APPswe, supporting further exploration of anatabine as a possible disease modifying agent for the treatment of AD.

## Introduction

Alzheimer’s disease (AD) is a progressive neurodegenerative disorder and is the most common form of dementia in the elderly, associated with deficits in social communication, memory and learning [1]. The pathological hallmarks of AD include intraneuronal formation of hyperphosphorylated tau protein aggregates forming neurofibrillary tangles and extracellular deposits of β-amyloid (Aβ) peptides, which constitute the main component of senile plaques [2]. Aβ peptides are derived from the processing of the amyloid precursor protein (APP) by β-secretase (BACE-1) and γ-secretase [3]. Post mortem examinations of AD brains also reveal other pathological abnormalities including loss of neurons and synapses, activation of glial cells and elevated levels of pro-inflammatory molecules. Inflammatory processes have been shown to contribute to the pathological accumulation of Aβ peptides by increasing APP expression and upregulating BACE-1 expression as well as some members of the γ-secretase complex [4]. Activated astrocytes and microglial cells are major mediators of neuroinflammation and produce elevated levels of pro-inflammatory cytokines which are known to ultimately activate nuclear factor kappa B (NFκB) and signal transducer and activator of transcription 3 (STAT3) signaling pathways [5]. NFκB and STAT3 are two transcription factors that regulate the expression of a large array of inflammatory genes [6] including cytokines such as IL1, IL-6, TNFα and inducible nitric oxide synthase (iNOS) and cycloxygenase-2 (COX-2) as well as some of the enzymes responsible for Aβ production [7,8] and mediates Aβ induced neurotoxicity [9]. Microglial activation and astrogliosis are therefore believed to play a pivotal role in the progression of AD. It has been suggested that nitric oxide produced via iNOS upregulation in activated microglia can promote neurodegeneration [10]. Interestingly, COX-2, a key enzyme in arachidonic acid metabolism mediating prostanoids production, has been found to be highly expressed in Aβ-burdened neurons which are thought to initiate neuroinflammation in AD [11]. COX-2 is also significantly expressed in activated microglia and astrocytes [12] and has been shown to be elevated in AD brain compared to healthy individuals [13,14]. Thus, suppression of NFκB and STAT3 pathways may represent an attractive approach for targeting neuroinflammation in AD.

Anatabine is a natural alkaloid found in plants of the Solanacea family (including eggplant, tomatoes and peppers). Anatabine has a chemical structure closely related to nicotine and is a full agonist of α7 and α4β2 nicotinic acetylcholine receptors (nAChR) but is a more potent α7 nAChR agonist than nicotine [15,16]. We have shown previously that anatabine displays some anti-inflammatory properties by reducing the activation of NFκB and STAT3 [17,18]. In the present study, we investigated the effects of a chronic oral treatment with 10 and 20 mg/Kg/Day of anatabine on the pathological deposition of Aβ, neuroinflammation and behavior in a transgenic mouse model of AD (Tg PS1/APPswe). We selected a dosage of 20 mg/Kg/Day based on the study by Caturegli and colleagues [19] reporting an anti-inflammatory activity of anatabine at this dosage and of our previous findings showing that at this dose, anatabine lowers neuroinflammation in various mouse models of CNS inflammation [18,20,21]. In addition, we elected to test the impact of a 10 mg/Kg/Day dose of anatabine to determine whether a lower dose of anatabine can affect AD-like pathology and the behavior of Tg PS1/APPswe mice. In order to model therapeutic intervention in mild to moderate AD rather than a prophylactic approach, anatabine treatment was initiated in 10 month-old mice, an age at which they already present significant Aβ deposits and cognitive impairments [22].

## Materials and Methods

### Animals and treatments

All the experiments involving mice were performed in the Association for Assessment and Accreditation of Laboratory Animal Care International (AAALAC) accredited vivarium of the Roskamp Institute and this particular study was approved by the Institutional Animal Care and Use Committee of the Roskamp Institute (IACUC protocol # R44). Mice were singly housed in ventilated cages on standardized rodent bedding in a specific pathogen-free environment. Transgenic mice over-expressing the human APP695 with the Swedish mutation K595N/M596L and the presenilin-1 mutation M146L (Tg PS1/APPswe) [22] and their control wild-type littermates were used in this study. All the transgenic mice used in this study were heterozygous for the transgenes. Anatabine base was provided by Rock Creek Pharmaceuticals (FL, USA) and was administered in the drinking water of the mice at a dosage of 10 or 20 mg/Kg of body weight/day while placebo treated mice received regular drinking water. Anatabine was administered at a dosage of 58 μg/ml of drinking water equivalent to an approximate oral uptake of 10mg/Kg/Day and 116 μg/ml for the 20mg/Kg/Day based on the fact that B6/SJL mice drink on average 6ml per day for an average body weight of 35 g [23]. Treatment group included: placebo, receiving regular drinking water (wild-type n = 9, Tg PS1/APPswe n = 8), anatabine at a dosage of 10mg/Kg/Day (wild-type n = 10, Tg PS1/APPswe n = 8) and at a dosage of 20mg/Kg/Day (wild-type n = 10, Tg PS1/APPswe n = 7). Each group included an equal number of male and female mice. The treatments were initiated in 10 month-old mice and lasted for 6 and half months. During the treatment period mice were observed on a daily basis and weighed once every other week to ensure no loss of weight occurred following the anatabine treatment. Mice were subjected to a battery of behavioral tests (at different time points in the study as described below) and humanely euthanized at the completion of the study at the age of 16.5 months. From each mouse, one brain hemisphere was fixed in 4% paraformaldehyde for pathological evaluation whereas the other hemisphere was snap frozen in liquid nitrogen and used for quantitative real time polymerase chain reaction (RT-qPCR).

### Elevated Plus Maze (EPM)

The EPM was used to assess anxiety like behavior in mice as described [24]. Briefly, 10.5 month-old mice were placed in the center of the EPM apparatus facing the open arm and left to explore the elevated plus maze for 10 minutes while 12.5 month-old mice were tested for 5 minutes. After each trial, the apparatus was wiped with 70% ethanol and dried. The time spent in the open and closed arms along with number of closed and open arm entries and distance travelled in each arm were determined using a computerized video tracking system (Ethovision XT 8.5 Software, Noldus Information Technology, Wageningen, Netherlands). Mice were evaluated in the EPM after 15 days and 2.5 months of treatment with anatabine.

### Social interaction and social recognition memory test

The three-chamber test known as the “Crawley's sociability and preference for social novelty protocol” was used to assess sociability and social memory [25]. The apparatus consists of three compartments made of clear plexiglas, each separated by side-doors. The test was conducted in a rectangular compartment that included a middle chamber with two side doors leading into two separate chambers (left and right), each chamber contained a steel cage enclosure allowing sensory interaction (smell, sight, and sound) but preventing direct physical contact, eliminating fighting or aggressive behaviors. To begin the test, the experimental mouse was placed in the middle chamber for 5 minutes of habituation with both side doors closed. After 5 minutes of habituation period, an unfamiliar mouse (Stranger 1) was introduced in the left chamber held behind the steel cage enclosure, and the experimental mouse was left to freely explore the chambers for 10 minutes. Social interaction was determined by measuring the length of time spent by the test mouse exploring the chamber holding the unfamiliar Stranger 1 vs. the empty chamber. To measure the social memory (or social novelty) a new unfamiliar mouse (Stranger 2) was placed in the steel enclosure in the previously empty chamber and the unfamiliar Stranger 1 was retained in the same chamber, and the experimental mouse was left to freely explore the chambers for 10 minutes. The amount of time spent by the test mouse for exploring the chamber containing the familiar Stranger 1 and the novel unfamiliar Stranger 2 mouse was measured to determine the preference of the test mouse for Stranger 1 and Stranger 2 as an indication for social memory or social novelty. Mouse activity was recorded with the aid of video tracking software (EthoVision XT 8.5, Noldus Information Technology, Wageningen, Netherlands). The apparatus was cleaned with 70% ethanol between each test mouse.

### Immunohistochemistry and image analyses

One brain hemisphere was fixed in 4% paraformaldehyde for 48 hours at 4°C, paraffin embedded and sectioned into 6μm sagittal sections using a microtome as we previously described [20]. Prior to staining, sections were deparaffinized in xylene (2 x 5 minutes), followed by hydration in an ethanol gradient (2 x 5 minutes in 100%, 95% and 70%) and finally rinsed in PBS. Heat induced antigen retrieval for Iba-1, phospho-STAT3, phospho-p65 NFκB and CD45 (cluster of differentiation 45) was performed by immersing the sections in 90–95°C preheated 10mM citrate buffer at pH 6.1 (DAKO, CA, USA) for 7 minutes. Slides were then allowed to cool for another 15 minutes, followed by sequential rinsing in PBS. The sections were immersed in 3% H_2_O_2_ for 30 minutes to quench the endogenous peroxide activity. Sections were rinsed with PBS and incubated with blocking buffer (Protein Block Serum-free Solution, Dako, CA, USA) for 45 minutes in a humidifying chamber. Brain sections were stained with the following antibodies diluted with Dako antibody diluent (CA, USA): 1:750 dilution monoclonal anti-β-amyloid antibody 4G8 (Covance, MA, USA), 1: 5,000 dilution polyclonal goat anti-Iba1 (ionized calcium binding adaptor molecule 1) (Abcam, MA, USA), 1:300 rabbit monoclonal anti-phospho-STAT3 (Tyr705) (Cell Signaling Technology Inc, MA, USA), 1:600 rabbit polyclonal anti-phospho-p65 NFκB (S536) (Santa Cruz, CA, USA) and 1:1000 rat monoclonal anti-mouse CD45 (Serotac, Raleigh, NC, USA). The diluted antibodies were applied onto the sections and incubated overnight at 4°C in a humidified chamber and were detected using Vectastain ABC (avidin-biotin-peroxidase complex) Elite kits (Vector Laboratories, CA, USA). Labeling was revealed by incubating sections in 0.01M PBS containing 0.05% 3,3'-diaminobenzidine (DAB) (Sigma, MO) and 0.015% of H_2_O_2_ (pH 7.2). After immunostaining with phospho-p65 NFκB and phospho-STAT3, sections were counterstained with Congo red, according to the recommendation of the manufacturer (Sigma-Aldrich, MO, USA) to reveal β-amyloid deposits. For each mouse, 4 to 6 non-consecutive randomly selected sections containing the hippocampus and cortex were used for each immunostain. For each brain section analyzed, 8 to 10 randomly selected pictures in the cortex and 5–6 pictures covering the entire hippocampal area were taken using a 20X objective for 4G8 and a 40X objective for Iba1 and phospho-STAT3 immunostaining using a digital camera connected to an Olympus BX60 microscope (Olympus, PA, USA). 4G8, Iba1 and phospho-STAT3 burden were quantified using the Image-Pro Plus software (Media Cybernetics, MD, USA) as we previously described [20]. For the quantification of phospho-p65 NFκB and CD45 burden associated with β-amyloid deposits, 4 to 6 brain sections per mouse were analyzed and 15–20 pictures containing β-amyloid plaques were randomly selected in the cortex using a 40X objective as described above. Phospho-p65 NFκB burden associated with β-amyloid deposits was quantified by image analysis using the Image-Pro Plus software (Media Cybernetics, MD, USA) whereas CD45 burden associated with β-amyloid deposits was quantified using the ImageJ morphometric image analysis software as previously described [26]. To ensure antibody specificity, negative control slides were incubated in the absence of primary antibodies for each antibody stain mentioned above. An average value of Aβ (4G8), Iba1, phospho-STAT3, phospho-p65 NFκB and CD45 burden (representing the area of immunoreactivity expressed as a percentage of the brain area examined) was calculated and an average burden determined for each treatment group.

### RNA extraction, cDNA synthesis, and quantitative PCR

Brain tissue was homogenized using a 1 ml Dounce homogenizer and total RNA was extracted using TRIzol, according to the manufacturer’s recommendations (Invitrogen, CA, USA). The purity and quantity of the extracted total RNA were tested on agarose gels and spectrophotometrically at 260 nm and 280 nm. All RNA samples had an A260/280 absorbance ratio between 1.9 and 2.1. 2000 ng of total RNA was used for cDNA synthesis by using the Transcription first stand cDNA synthesis kit (Roche, USA) with random hexamer primers according to the manufacturer’s protocol. TaqMan gene expression assays (Applied Biosystems, NY, USA) were used for the following genes: β-site APP Cleaving Enzyme 1 (*Bace1*) (Mm00478664_m1), inducible Nitric Oxide Synthase (*iNOS*) (Mm00440485_m1), Cyclooxygenase 2 (*Cox-2*) (Mm00478374_m1) and Glyceraldehyde-3-phosphate dehydrogenase (*Gapdh*) (Mm99999915). TaqMan real-time PCR was performed using primers and probe sets from Applied Biosystems. All the reactions were done in duplicate. The threshold cycle number was used to calculate the relative expression levels of the genes of interest. Amplification was performed by using the QuantStudio 7 Flex Real-Time PCR System (Applied Biosystems, USA) with parameters of 95°C for 20 sec followed by 40 cycle of 95°C for 1 sec denaturation and 60°C for 20 sec annealing and extension. Data analyses were performed using QuantStudio 6 and 7 Flex Software (Applied Biosystems, USA) to measure the threshold cycle (Ct) for each reaction. The mean Ct value was established by using duplicate Ct values and analysis was completed by using the ΔΔCt method [27]. Data were normalized to *Gapdh* messenger RNA (mRNA) levels and expressed as a relative fold change compared to control samples (wild-type placebo).

### Statistical analysis

Results are expressed as mean ± SEM. Statistical analyses were performed using SPSS v12.0.1 for Windows. Data were first examined for assumption of normality using the Shapiro-Wilk statistic and for homogeneity of variance using the Levene's test. Statistical significance was determined by Student’s t-test, univariate or repeated measures analysis of variance (ANOVA) where appropriate followed by post-hoc comparisons with the Bonferroni method. For data not satisfying assumptions of normality and homogeneity of variance, a nonparametric Mann-Whitney test was used. P-values < 0.05 were considered significant.

## Results

### Effects of anatabine on the behavior of Tg PS1/APPswe mice

**Fig 1:**
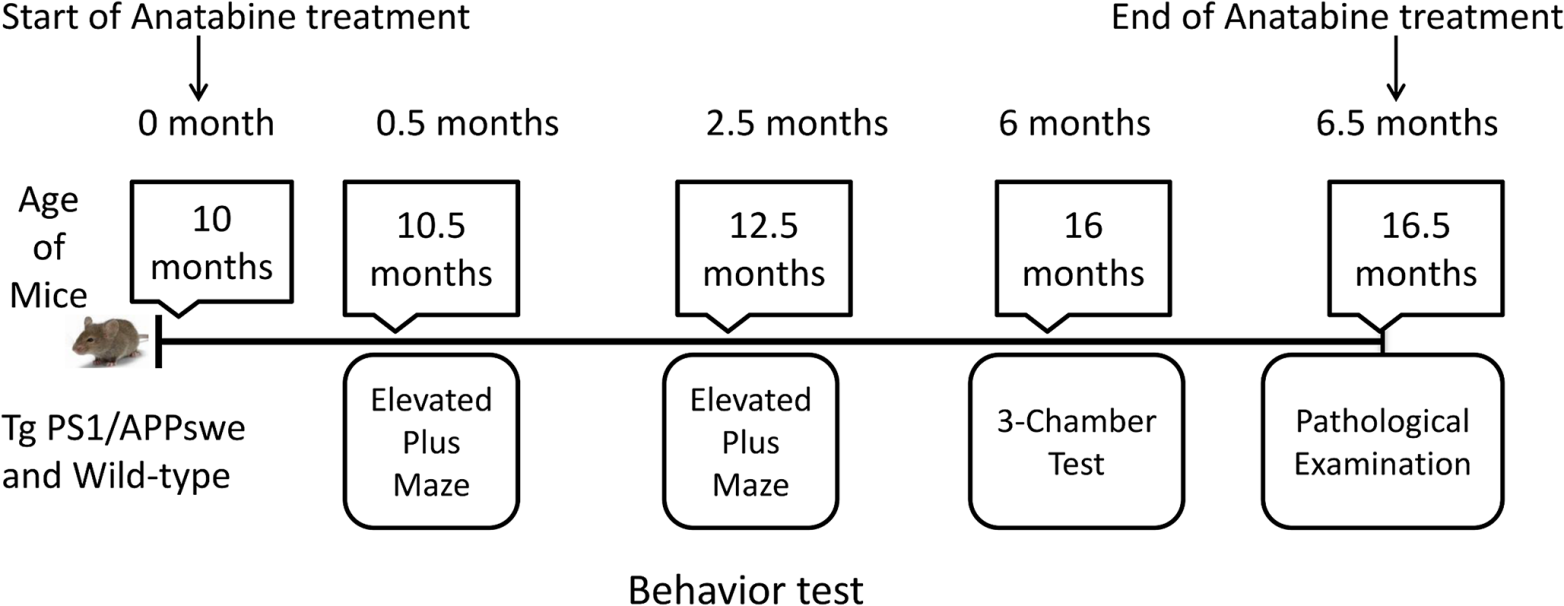
Timeline of anatabine treatment and behavior tests performed in Tg PS1/APPswe mice and their control wild-type littermate. Oral anatabine treatment with 10mg/Kg/day or 20mg/Kg/Day was started in 10 months old Tg PS1/APPswe mice and control wild-type littermates and lasted for 6 and half months. During the course of treatment, a battery of behavior tasks was performed at the time points indicated above. Possible effects of the anatabine treatment on anxiety in mice were evaluated using the elevated plus maze; 0.5 month and 2.5 months after the initiation of the anatabine treatment (age of mice 10.5 and 12.5 months respectively). To evaluate the effects of anatabine on social interaction and social memory, the 3-chamber test was used 6 months after the treatment initiation (age of mice 16 months). At the end of study mice were humanely euthanized at the age of 16.5 months for pathological evaluations.

**Fig 2:**
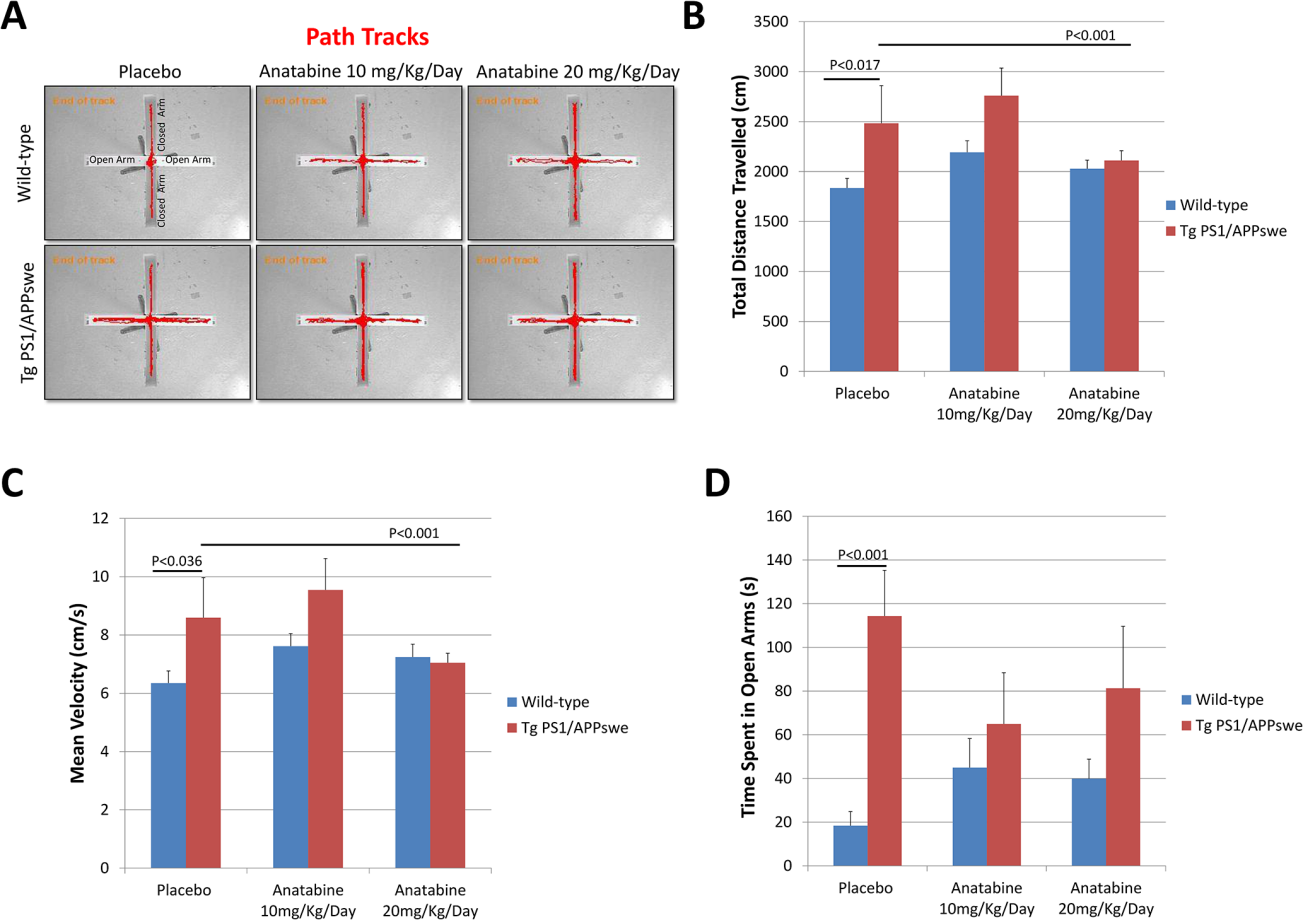
Anatabine reduces hyperactivity and disinhibition in 10.5 month-old Tg PS1/APPswe mice. 10.5 month-old Tg PS1/APPswe and their control littermates tested in EPM after treatment duration of 0.5 months with anatabine. A) Representative path tracks in the closed and open arms of the elevated plus maze by 10.5 months old Tg PS1/APPswe and their control wild-type littermate receiving regular drinking water (placebo) and anatabine at a dosage of 10 and 20 mg/Kg/Day in their drinking water are shown. B and C) The histogram represents the total distance travelled and mean velocity of Tg PS1/APPswe mice and their control wild-type littermates receiving regular drinking water (placebo) and anatabine at a dosage of 10 or 20 mg/Kg/Day. ANOVA revealed a significant main effect of the genotype for the total distance travelled (P<0.009) and mean velocity (P<0.036). Post-hoc analyses show that Tg PS1/APPswe placebo mice travelled more distance compared to wild-type placebo (P<0.017) and showed greater velocity compared to their control wild-type placebo mice (P<0.036). A significant reduction in hyperactivity was observed in Tg PS1/APPswe mice receiving anatabine at a dosage of 20 mg/Kg/Day compared to Tg PS1/APPswe mice receiving regular drinking water (placebo) (P<0.001). D) The histogram represents the average amount of time spent by Tg PS1/APPswe and wild-type mice in the open arms of the elevated plus maze. ANOVA reveals a significant main effect of the genotype (P<0.002) as well as an interactive term between genotype and anatabine (P<0.018) for the time spent in the open arm. Post-hoc comparisons show that Tg PS1/APPswe placebo mice spent significantly more time in the open arm than wild-type control mice (P<0.001). Tg PS1/APPswe mice receiving anatabine at a dosage of 10 and 20 mg/Kg/Day spent a similar amount of time (P>0.05) in the opens arms as their control wild-type littermates. The number of individuals (n) tested in three groups were I) Mice receiving regular drinking water (placebo): wild-type (n = 8) and Tg PS1/APPswe (n = 8); II) Mice receiving anatabine at a dosage of 10mg/Kg/Day: wild-type (n = 10) and Tg PS1/APPswe (n = 8) and III) Mice receiving anatabine at a dosage of 20mg/Kg/Day: wild-type (n = 10) and Tg PS1/APPswe (n = 8).

**Fig 3:**
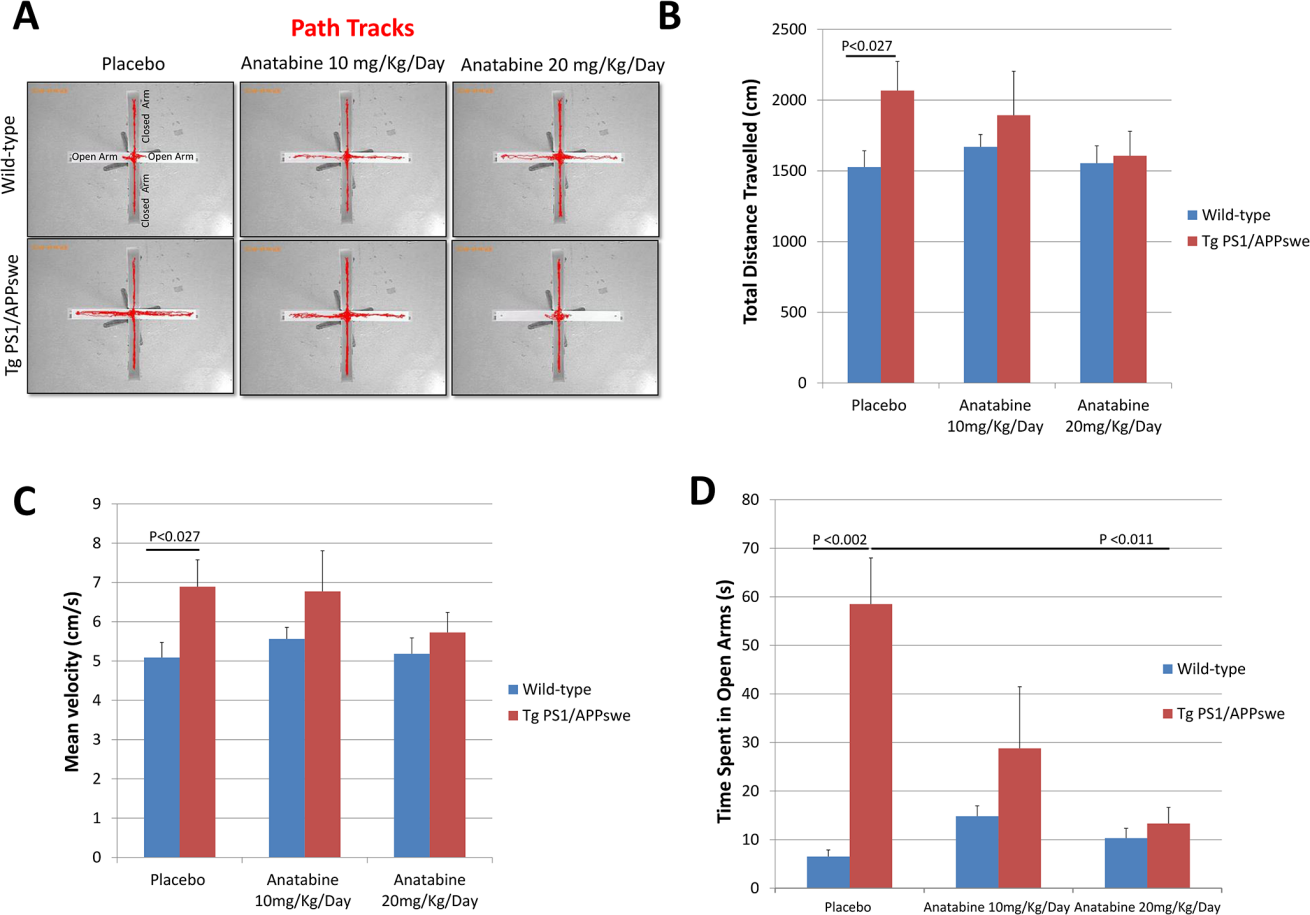
Anatabine reduces hyperactivity and disinhibition in 12.5 month-old Tg PS1/APPswe mice. 12.5 month-old Tg PS1/APPswe and their control littermates tested in EPM after treatment duration of 2.5 months with anatabine. A) Representative path tracks in the closed and open arms of the elevated plus maze by 12.5 months old Tg PS1/APPswe and their control wild-type littermate receiving regular drinking water (placebo) and anatabine at a dosage of 10 and 20 mg/Kg/Day in their drinking water are shown. B and C) The histogram represents the total distance travelled and mean velocity of Tg PS1/APPswe mice and their control wild-type littermates receiving regular drinking water (placebo) and anatabine at a dosage of 10 or 20 mg/Kg/Day. ANOVA showed a significant main effect of the genotype for the distance travelled (P<0.008) and mean velocity (P<0.008). Post-hoc comparisons show that Tg PS1/APPswe placebo mice were significantly different compared to their wild-type littermate mice for distance travelled (P<0.027) and velocity (P<0.027). Tg PS1/APPswe mice receiving anatabine at a dosage of 10 or 20 mg/Kg/Day were not significantly different from wild-type mice (P>0.05). D) The histogram represents the average amount of time spent by Tg PS1/APPswe and wild-type mice in open arms of the elevated plus maze. ANOVA reveals a significant main effect of the genotype (P<0.003) and an interaction between genotype and anatabine (P<0.029) for the time spent in the open arm. Post-hoc comparison shows that Tg PS1/APPswe placebo mice spent significantly more time in the open arms than wild-type control mice (P<0.002). Tg PS1/APPswe mice receiving anatabine at a dosage of 20 mg/Kg/Day spent significantly less time in the open arms of the EPM compared to Tg PS1/APPswe receiving regular drinking water (placebo) (P<0.011). Overall, anatabine treated Tg PS1/APPswe and wild-type littermate mice spent the same amount of time in the open arms (P>0.183 and P>0.683). The number of individuals (n) tested in three groups were I) Mice receiving regular drinking water (placebo): wild-type (n = 8) and Tg PS1/APPswe (n = 8); II) Mice receiving anatabine at a dosage of 10mg/Kg/Day: wild-type (n = 10) and Tg PS1/APPswe (n = 8) and III) Mice receiving anatabine at a dosage of 20mg/Kg/Day: wild-type (n = 10) and Tg PS1/APPswe (n = 8).

Transgenic mouse models of AD overexpressing Aβ peptides generally show greater locomotor activity and disinhibition in the elevated plus maze compared to non-transgenic mice, suggesting hyperactivity and a lower level of anxiety [28–30]. To assess the possible effect of anatabine on locomotor activity and anxiety like behavior in Tg PS1/APPswe and their control wild-type littermates, mice were tested in the elevated plus maze (Fig 1). First when tested at the age of 10.5 months of age (15 days in anatabine treatment), Tg PS1/APPswe receiving regular drinking water (placebo) showed increased locomotor activity (as measured by distance travelled and velocity) compared to wild-type mice as expected (Fig 2B and 2C). Interestingly, the hyperactive behavior of Tg PS1/APPswe mice was suppressed with the anatabine treatment at a dosage of 20 mg/Kg/Day (Fig 2B and 2C). With regard to disinhibition, as expected Tg PS1/APPswe receiving regular drinking water (placebo) spent more time in the opens arms and less time in the closed arms of the elevated plus maze compared to control wild-type littermates (Figs 2D and 3D). The disinhibition affecting Tg PS1/APPswe mice was suppressed following treatment with anatabine at a dosage of either 10 or 20 mg/Kg/Day at both the time points (Figs 2D and 3D). Wild-type mice treated with anatabine at a dosage of 10 and 20 mg/Kg/Day showed a trend for spending more time in the opens arms of the elevated plus maze but this effect was not statistically significant (Figs 2D and3D).

**Fig 4:**
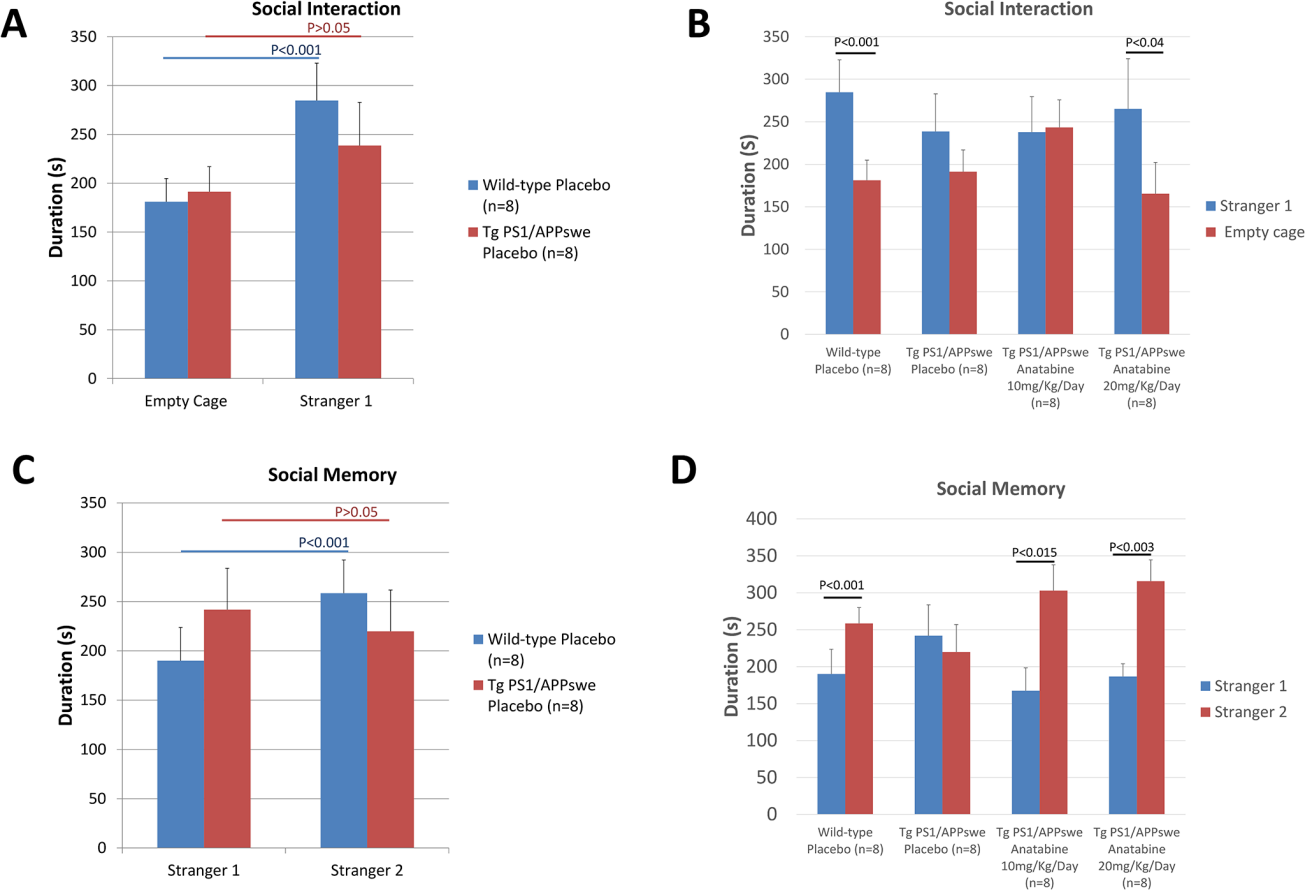
Anatabine improves social interaction and social memory in Tg PS1/APPswe mice. A) The histogram represents the mean time spent ± SEM by Tg PS1/APPswe and their control wild-type littermates mouse receiving regular drinking water in the chamber containing the Stranger1 and the empty cage for the social interaction phase of 3-chamber test. B) The histogram represents the time spent ± SEM by Tg PS1/APPswe receiving regular drinking water or anatabine at a dosage of 10 and 20 mg/Kg/Day in the chamber containing the Stranger 1 and the empty cage for the social interaction phase of 3-chamber test. Tg PS1/APPswe mice receiving regular drinking water spent the same amount of time in the chamber containing the stranger 1 and the empty cage (P>0.05). Tg PS1/APPswe mice receiving anatabine at a dosage of 20mg/Kg/Day spent significantly more time exploring the chamber containing the Stranger1 than the chamber containing the empty cage (P<0.04). C) The histogram represents the time spent ± SEM by Tg PS1/APPswe and their control wild-type littermate mouse in the chamber containing the Stranger1 and the Stranger 2 for the social memory phase of 3-chamber test. D) The histogram represents the time spent ± SEM by Tg PS1/APPswe receiving regular drinking water or anatabine at a dosage of 10 and 20 mg/Kg/Day in the chamber containing the Stranger 1 and in the chamber containing the Stranger 2. Tg PS1/APPswe mice receiving regular drinking water showed no preference for social novelty, as they spent an equal amount of time in the chamber containing the Stranger 1 and in the chamber containing the Stranger 2 (P>0.05). Tg PS1/APPswe mice receiving anatabine at a dosage of 10 and 20 mg/Kg/Day spent significantly more time exploring the chamber containing the unfamiliar Stranger 2 than the chamber containing the previously encountered mouse (Stranger 1) (P<0.015 and P<0.003 respectively).

Profound alterations of social behavior have been described in transgenic mouse models of AD [31]; we therefore explored whether anatabine could affect social interaction and social memory in Tg PS1/APPswe mice using the 3-chamber test known as the Crawley's sociability and preference for social novelty protocol [25,32,33]. When exposed to the social interaction test, wild-type mice demonstrated sociability by spending more time in the chamber containing an unknown mouse (Stranger 1) than in the chamber containing an empty cage (Fig 4A). Unlike wild-type mice, Tg PS1/APPswe mice elicited social interaction deficits and spent an equal amount of time in the chamber containing the empty cage or the chamber containing the unfamiliar (Stranger 1) mouse (Fig 4A). Anatabine at a dosage of 20mg/Kg/Day restored sociability in Tg PS1/APPswe mice as Tg PS1/APPswe mice treated with anatabine spent significantly more time in the chamber containing the unfamiliar mouse (Stranger 1) and less time in the chamber containing the empty cage (Fig 4B). In the social memory phase of the 3-chamber test, wild-type mice spent more time in the chamber containing the novel unfamiliar mouse (Stranger 2) than in the chamber containing the previously encountered mouse (Stranger 1) showing social memory or preference for social novelty (Fig 4C). By contrast, Tg PS1/APPswe mice receiving regular drinking water showed no preference for the chamber containing the novel unfamiliar mouse (Stranger 2) suggesting a lack of short-term social memory (Fig 4C). However, anatabine significantly improved social memory in Tg PS1/APPswe mice (Fig 4D).

### Effect of anatabine on microgliosis in Tg PS1/APPswe mice

**Fig 5:**
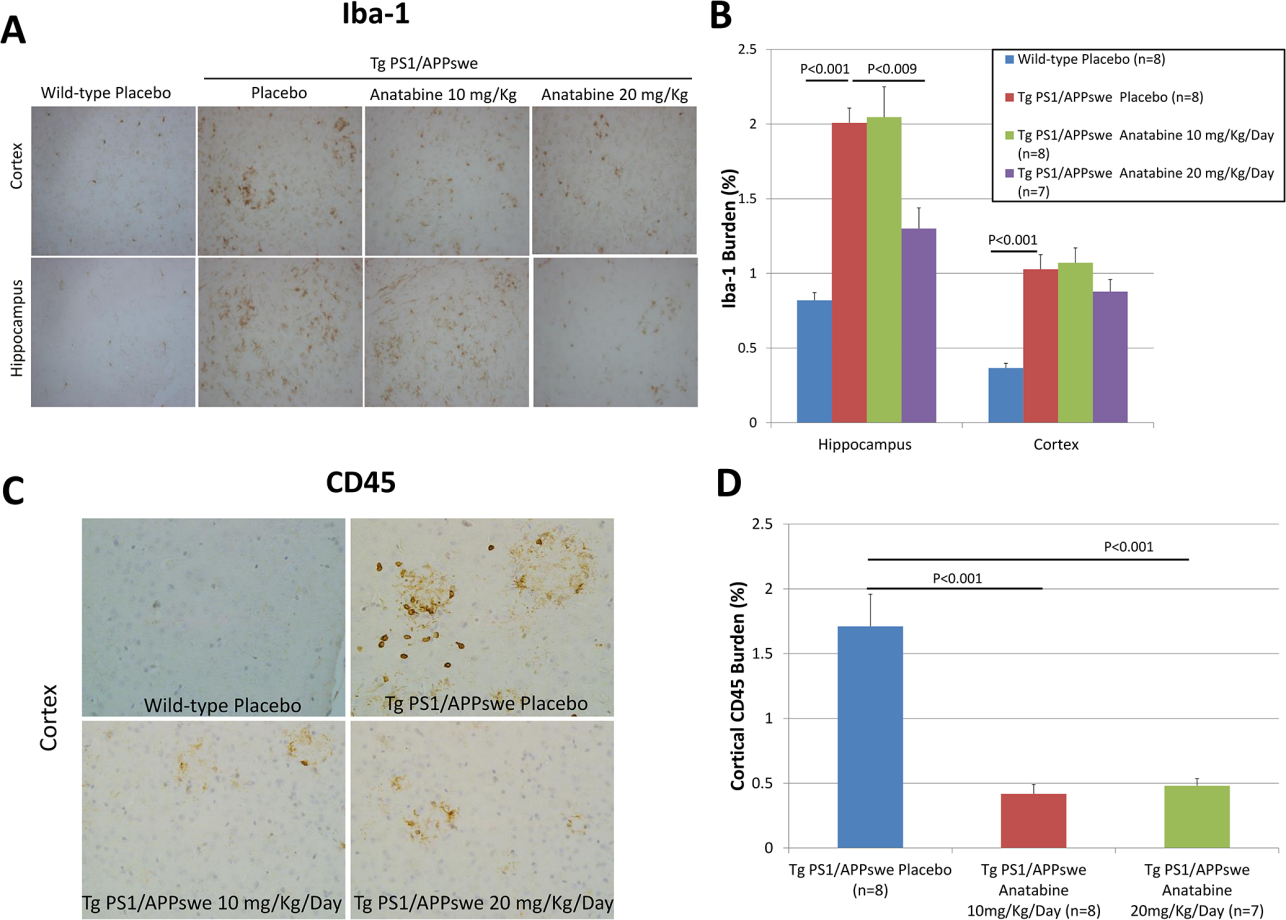
Anatabine reduces microgliosis in the brain of Tg PS1/APPswe mice. A) Representative 40X microscopic fields revealing Iba1 immunoreactive microglial cells in the hippocampus and the cortex of wild-type placebo and Tg PS1/APPswe receiving regular drinking water (placebo) and Tg PS1/APPswe mice receiving anatabine at a dosage of 10 and 20 mg/Kg/Day dissolved in their drinking water. Data is presented as mean±SEM. B) The histogram represents the average amount of Iba1 burden (expressed as a percentage of the brain area examined) quantified in the hippocampus and the cortex of wild-type receiving regular drinking water (placebo) and Tg PS1/APPswe receiving either regular drinking water (placebo) or anatabine at a dosage of 10 and 20 mg/Kg/Day dissolved in their drinking water. ANOVA reveals a significant main effect of the genotype on Iba1 burden in the hippocampus and cortex (P<0.001 and P<0.001). Post-hoc comparisons show a significant reduction in Iba1 burden in the hippocampus of Tg PS1/APPswe (P<0.009) treated with 20 mg/Kg/Day of anatabine compared to Tg PS1/APPswe receiving regular drinking water (placebo). C) Representative 40X microscopic fields revealing CD45 immunoreactive microglial/macrophage cells associated with β-amyloid plaques in the cortex of Tg PS1/APPswe receiving regular drinking water (placebo) and Tg PS1/APPswe mice receiving anatabine at a dosage of 10 and 20 mg/Kg/Day in their drinking water. D) The histogram represents the average amount of CD45 burden (expressed as a percentage of the brain area examined) quantified in the cortex of Tg PS1/APPswe receiving either regular drinking water (placebo) or anatabine at a dosage of 10 and 20 mg/Kg/Day dissolved in their drinking water. ANOVA shows a significant main effect of the anatabine treatment on CD45 burden (P<0.001). Post-hoc comparisons reveal significant differences between placebo and anatabine treated mice at a dosage of 10mg/Kg/Day (P<0.001) and 20 mg/Kg/Day (P<0.001).

It has been reported that microgliosis contributes to neurodegeneration in AD. Once activated, microglia produce inflammatory cytokines, free radicals and can generate Aβ [34]. We therefore explored the impact of anatabine treatment on microgliosis in the brains of Tg PS1/APPswe and their control wild-type littermates. Iba-1 protein is expressed by microglia and has been shown to be upregulated during microgliosis [35]. A significant increase in Iba-1 burden was observed in the cortex and hippocampus of Tg PS1/APPswe mice compared to their control wild-type littermates, suggesting increased Iba-1 immunopositive microglia in the brain of Tg PS1/APPswe mice (Fig 5A). Interestingly, Tg PS1/APPswe mice treated with anatabine at a dosage of 20 mg/Kg/Day, but not 10mg/Kg/day showed a significant reduction in Iba-1 burden in the hippocampus compared to untreated Tg PS1/APPswe mice (Fig 5A and 5B), suggesting that anatabine suppresses microgliosis in the brain of Tg PS1/APPswe mice. We further assessed the stage of activated microglia/macrophages in the cortex of Tg PS1/APPswe placebo mice using CD45 immunostaining. A significant increase in CD45 immunopositive microglia/macrophage was observed in the cortex of Tg PS1/APPswe mice compared to wild-type mice (CD45 immunonegative) (Fig 5C). However, we observed a significant reduction in CD45 immunopositive microglia/macrophage in Tg PS1/APPswe mice receiving anatabine at a dosage of 10 and 20 mg/Kg/Day compared to Tg PS1/APPswe mice receiving regular drinking water (Fig 5C and 5D).

### Effects of anatabine on STAT3 and NFκB activation in Tg PS1/APPswe brain

**Fig 6:**
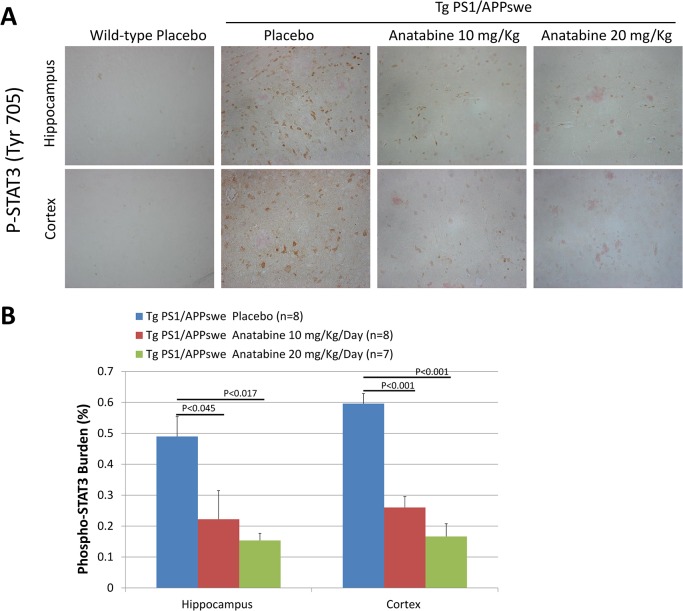
Anatabine reduces the amount of phosphorylated STAT3 immunopositive cells in the hippocampus and cortex of Tg PS1/APPswe mice. A) Representative 40X microscopic fields revealing the presence of phospho-STAT3 immunopositive cells around Congo red stained β-amyloid deposits in the hippocampus and cortex of wild-type mice and Tg PS1/APPswe receiving regular drinking water (placebo), and Tg PS1/APPswe mice receiving anatabine at a dosage of 10 and 20 mg/Kg/Day in their drinking water are shown. Data is presented as mean±SEM. B) The histogram represents the average amount of phospho-STAT3 burden (expressed as a percentage of the brain area examined) quantified in the hippocampus and the cortex of Tg PS1/APPswe mice receiving the regular drinking water (placebo) or anatabine at a dosage of 10 and 20 mg/Kg/Day. ANOVA shows a statistically significant main effect of the anatabine treatment on phospho-STAT3 burden associated with Aβ deposits (P<0.001). Post-hoc analyses reveal statistically significant differences in phospho-STAT3 burden in the hippocampus associated with β-amyloid burden (P<0.045 and P<0.017) and cortex (P<0.001 and P<0.001) between Tg PS1/APPswe placebo and anatabine treated 10 and 20 mg/Kg/Day Tg PS1/APPswe mice. (No immunoreactivity for phospho-STAT3 was observed in the hippocampus and the cortex of wild-type mice).

**Fig 7:**
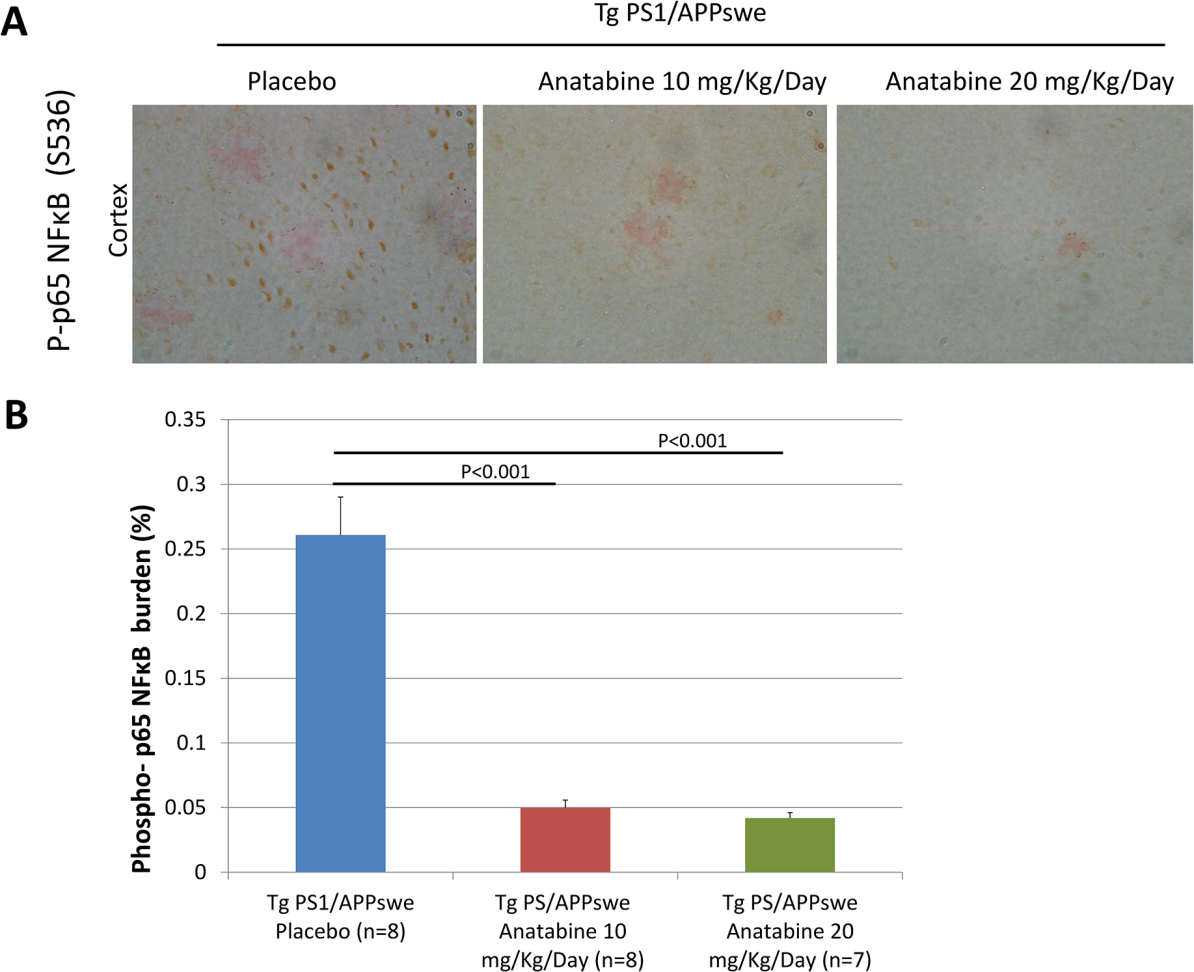
Anatabine reduces the amount of phosphorylated p65 NFκB immunopositive cells associated with β-amyloid deposits in the cortex of Tg PS1/APPswe mice. A) Representative 40X microscopic field revealing the presence of phosphorylated p65 NFκB immunopositive cells around Congo red stained β-amyloid deposits in the cortex of Tg PS1/APPswe mice receiving regular drinking water (placebo) or anatabine at a dosage of 10 and 20 mg/Kg/Day in their drinking water. Data is presented as mean±SEM. B) The histogram represents the average amount of phospho p65 NFκB burden (expressed as a percentage of the brain area examined) associated with β-amyloid deposits in the cortex Tg PS1/APPswe receiving regular drinking water (placebo) and the anatabine treatment at a dosage of 10 and 20 mg/Kg/Day. ANOVA shows a statistically significant main effect of the anatabine treatment on phosphorylated p65 NFκB immunoreactive cells associated with Aβ deposits (P<0.001). Post-hoc comparisons reveal statistically significant differences between Tg PS1/APPswe receiving regular drinking water (placebo) and anatabine at a dosage of 10 mg/Kg/Day (P<0.001) and 20 mg/Kg/Day (P<0.001) for the amount of p65 NFκB phosphorylation associated with β-amyloid burden.

We have previously shown that anatabine inhibits STAT3 and NFκB activation [18] resulting in decreased neuroinflammation in a mouse model of multiple sclerosis. Since STAT3 and NFκB play an important role in the activation of glial cells and also regulates BACE-1 mRNA expression [8,36], we explored the possible impact of the anatabine treatment on STAT3 and NFκB activation by monitoring STAT3 phosphorylation at Tyr705 and p65 NFκB phosphorylation at Ser536 in the brains of the Tg PS1/APPswe and their control wild-type mice. We observed an elevation of STAT3 phosphorylation in the hippocampus and cortex of Tg PS1/APPswe compared to their control wild-type littermates (Fig 6A). A significant reduction in STAT3 phosphorylation was observed in the hippocampus and cortex of Tg PS1/APPswe mice treated with 10 or 20 mg/Kg/Day of anatabine compared to Tg PS1/APPswe receiving regular drinking water (Fig 6B) showing that anatabine prevents STAT3 activation in the brain of Tg PS1/APPswe mice. We also observed elevation of NFκB activation in the vicinity of Aβ deposits in the brain of Tg PS1/APPswe mice (Fig 7A). Interestingly, we found a significant reduction in the expression of amyloid plaque associated phospho-p65 NFκB immunopositive cells in Tg PS1/APPswe mice treated with anatabine at either 10 or 20 mg/Kg/Day (Fig 7B) showing that anatabine prevents NFκB activation in the brain of Tg PS1/APPswe mice.

### Effects of anatabine on brain Aβ burden and *Bace1* mRNA expression

**Fig 8:**
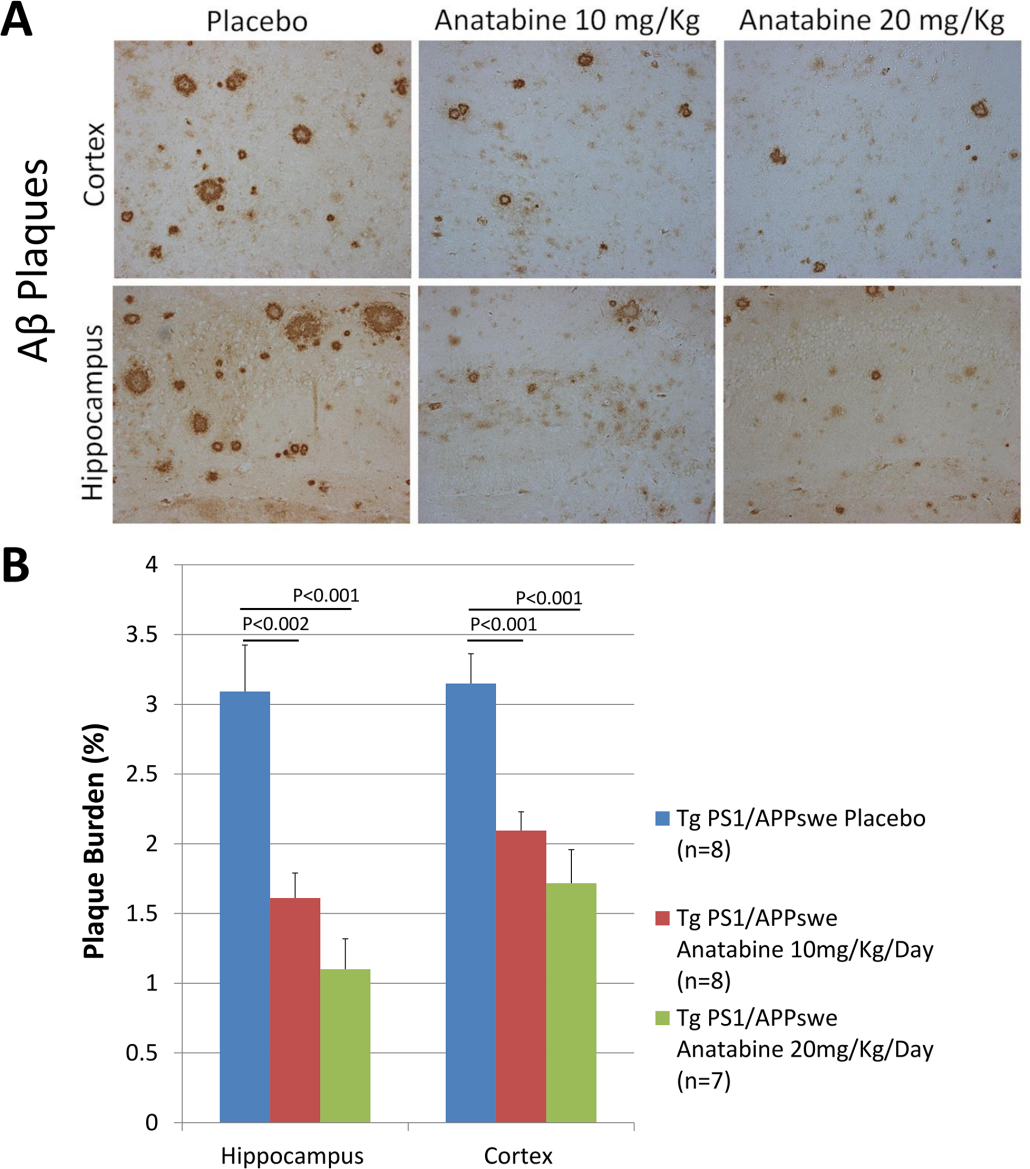
Anatabine reduces β-amyloidosis in the hippocampus and the cortex of Tg PS1/APPswe mice. A) Representative 20X microscopic fields showing β-amyloid deposits (4G8 immunostaining) in the cortex and hippocampus of Tg PS1/APPswe receiving regular drinking water (placebo) and anatabine at a dosage of 10 and 20 mg/Kg/Day dissolved in their drinking water are shown. Data is presented as mean±SEM. B) The histogram represents the average amount of 4G8 burden quantified in the hippocampus and cortex of Tg PS1/APPswe mice. ANOVA shows a statistically significant main effect of the anatabine treatment (P<0.001) on β-amyloid plaque burden. Post-hoc analyses reveal statistically significant differences in β-amyloid burden in the hippocampus (P<0.002 and P<0.001) and cortex (P<0.001 and P<0.001) between Tg PS1/APPswe mice receiving regular drinking water (placebo) and Tg PS1/APPswe receiving anatabine at a dosage of 10 and 20 mg/Kg/Day dissolved in their drinking water.

**Fig 9:**
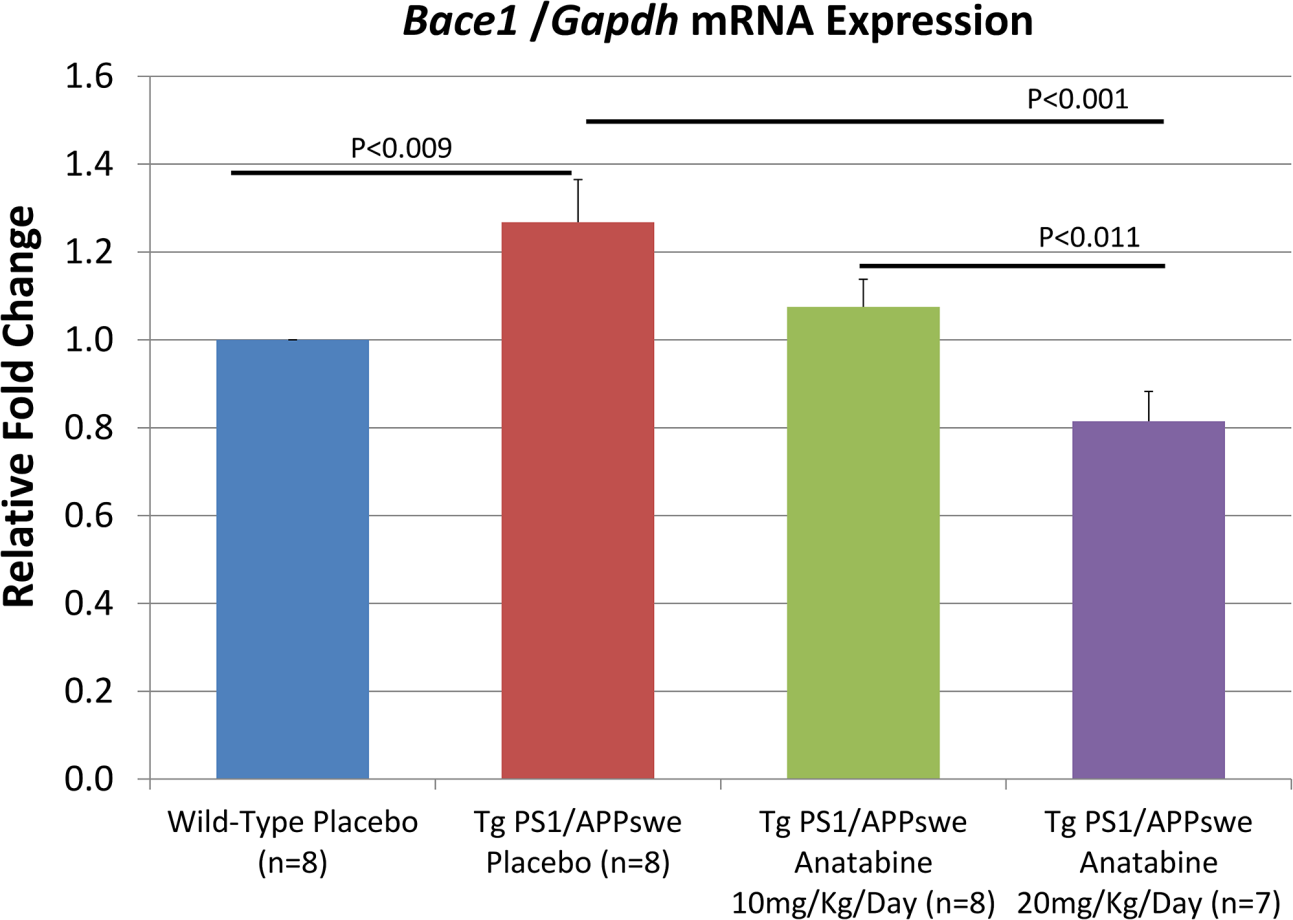
Anatabine reduces *Bace*1 mRNA expression in brain of Tg PS1/APPswe mice. The histogram represents quantitative results from RT-qPCR analysis calculated using the Delta-Delta Ct Method. The means ± SEM *Bace1* mRNA levels are expressed as a relative fold change compared to control (wild-type placebo). ANOVA shows a significant main effect of the genotype and treatments (P<0.009 and P<0.001) on *Bace1* transcription. Post-hoc analyses reveal a significant difference in *Bace1* mRNA levels between wild-type and Tg PS1/APPswe placebo mice (P<0.009) showing an upregulation of *Bace1* mRNA in Tg PS1/APPswe mice compared to wild-type littermates. A significant reduction in *Bace1* transcription was observed in Tg PS1/APPswe treated with 20 mg/Kg/Day of anatabine in their drinking water (P<0.001).

As anatabine reduces STAT3 and NFκB activation in the brains of Tg PS1/APPswe mice and since both NFκB and STAT3 have been shown to regulate *Bace1* expression [7,8,36], we therefore explored whether the Aβ burden and *Bace1* expression were impacted by the anatabine treatment in Tg PS1/APPswe mice. We observed that anatabine reduces brain Aβ burden both in the cortex and the hippocampus of Tg PS1/APPswe mice using immunostaining with the antibody 4G8 which recognizes Aβ (Fig 8A and 8B). Additionally, we found that *Bace1* mRNA expression is significantly increased in the brain of Tg PS1/APPswe mice compared to wild-type littermates, whereas a significant reduction in *Bace1* mRNA levels is observed in Tg PS1/APPswe receiving 20 mg/Kg/Day of anatabine in their drinking water showing that at this dosage anatabine can mitigate the upregulation of *Bace1* expression in Tg PS1/APPswe mice (Fig 9).

### Effect of anatabine on *Cox-2* and *iNOS* mRNA expression

**Fig 10:**
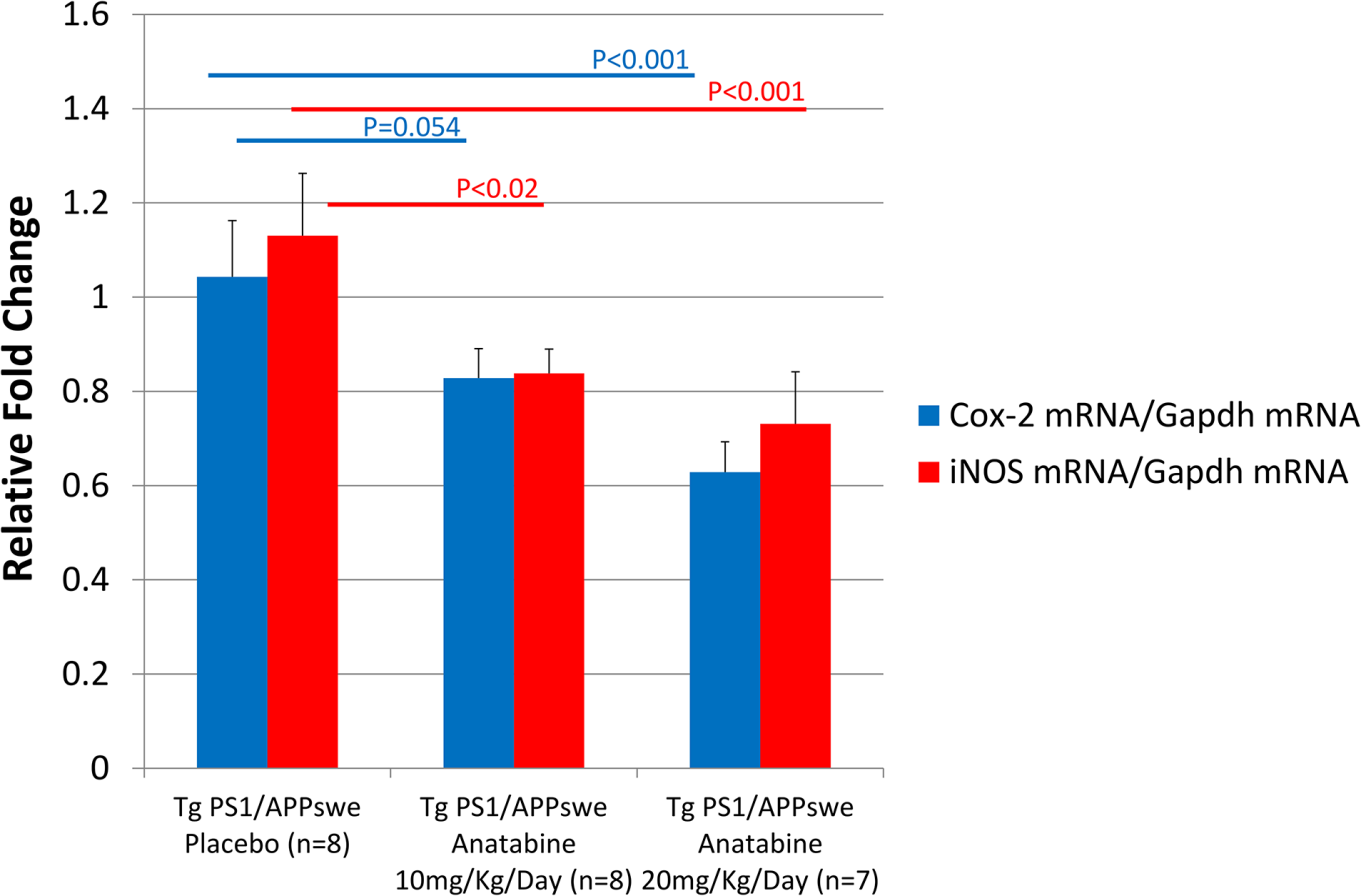
Anatabine reduces *Cox-2* and *iNOS* mRNA expression in brain of Tg PS1/APPswe mice. The histogram represents quantitative results from RT-qPCR analysis calculated using the Delta-Delta Ct Method. The means ± SEM *Cox*-2 and *iNOS* mRNA levels are expressed as relative fold change compared to control (wild-type placebo). ANOVA shows a significant main effect of the treatments on *Cox-2* and *iNOS* transcription (P<0.001). Post-hoc analyses reveal a significant difference in *Cox*-*2* and *iNOS* mRNA levels between Tg PS1/APPswe placebo and anatabine treated mice 10 mg/Kg/Day (P<0.02 for *iNOS*) and 20 mg/Kg/Day (P<0.001 for *iNOS* and P<0.001 for *COX-2*).

Since COX-2 and iNOS transcription are regulated by NFκB and STAT3 [6,37–39] and since both COX-2 and iNOS contribute to neuroinflammatory processes in AD [5], we also investigated whether *Cox-2* and *iNOS* mRNA expression were affected by anatabine in the brains of Tg PS1/APPswe mice. We found a significant reduction in *Cox-2* mRNA expression in Tg PS1/APPswe mice treated with anatabine at a dosage of 20 mg/Kg/Day (Fig 10). A dose dependent suppression of *iNOS* transcription was observed in Tg PS1/APPswe mice treated with anatabine at a dosage of 10 or 20 mg/Kg/Day compared to untreated Tg PS1/APPswe mice (Fig 10).

## Discussion

In this study, we evaluated the impact of a chronic oral treatment with anatabine on AD like pathology and behavioral deficits elicited by a transgenic mouse model of AD overproducing Aβ peptides. The anatabine treatment was initiated in 10 month-old Tg PS1/APPswe when significant Aβ deposition and cognitive impairment are present, to mimic an active treatment in AD. Our data reveal that Tg PS1/APPswe mice treated orally with anatabine in their drinking water show a significant reduction in Aβ deposition in the hippocampus and the cortex compared to Tg PS1/APPswe receiving regular drinking water. The reduction of brain Aβ burden was also accompanied by a significant decrease in Iba1 in the hippocampus suggesting that anatabine is reducing microgliosis. Under physiological conditions, quiescent microglia express a low level of CD45 whereas activated microglia express high level of CD45 and are therefore phenotypically similar to peripheral macrophages. Interestingly, it has been found that the majority of CD45 immunopositive cells in the CNS of Tg PS1/APPswe mice are resident microglia and not macrophages [40,41] suggesting that CD45 can represent a marker of activated microglia in the CNS of Tg PS1/APPswe. We observed a reduction in CD45 immunoreactive microglial/macrophage cells in the brain parenchyma of Tg PS1/APPswe mice further suggesting that anatabine is reducing microglial activation. This is further substantiated by the fact that the activation of NFκB and STAT3 in the vicinity of Aβ deposits is also significantly reduced in anatabine treated Tg PS1/APPswe mice showing overall that anatabine is reducing neuroinflammation in Tg PS1/APPswe mice. The transcription factors NFκB and STAT3 play a critical role in the propagation of inflammatory reactions within the CNS and notably in the activation of glial cells [34]. NFκB and STAT3 are also known to regulate the expression of BACE-1 as well as some members of the γ-secretase complex which are responsible for the production of Aβ peptides [4]. We therefore evaluated the possible impact of the anatabine treatment on *iNOS* and *Cox-2* mRNA levels which are known to be elevated in activated glial cells [42] and regulated in an NFκB and STAT3 dependent manner [6,37–39]. We found that both *iNOS* and *Cox-2* mRNA levels were significantly reduced in the brains of Tg PS1/APPswe mice treated with anatabine compared to Tg PS1/APPswe receiving regular drinking water, further illustrating the mitigation of neuroinflammation by anatabine. The reduction in *iNOS* expression conferred by anatabine may have also additional beneficial consequences. For instance iNOS has been shown to induce the nitration of Aβ resulting in an acceleration of its aggregation both *in vitro* and *in vivo* [43] and affecting Aβ deposition and cognitive dysfunction in Tg PS1/APPswe mice. The reduction in *iNOS* induced by anatabine in Tg PS1/APPswe mice may have also contributed to the reduction in Aβ deposition observed following treatment with anatabine. Besides its involvement in inflammatory reactions, *Cox-2* has also been shown to contribute to memory impairments induced by Aβ as COX-2 inhibition restored memory in a transgenic mouse model of AD and prevented the impairment of long term potentiation induced by Aβ [44]. We found that *Bace1* mRNA expression was also significantly reduced in the brain of Tg PS1/APPswe mice treated with anatabine which is also consistent with a reduction of NFκB and STAT3 signaling following treatment with anatabine and could have contributed to the reduction of Aβ deposition observed following anatabine treatment.

The beneficial effects of anatabine on neuroinflammation and Aβ accumulation were also associated with some behavioral improvements in Tg PS1/APPswe mice. However, we did not observed any beneficial effect of the anatabine treatment on spatial learning abilities in Tg PS1/APPswe mice as assessed by the radial arm water maze (RAWM) and Morris water maze (MWM) (S1 and S2 Figs). However, we observed that disinhibition, which affects several mouse models of AD overexpressing Aβ peptides [28,30,45], was alleviated by the anatabine treatment in Tg PS1/APPswe mice while a trend for a reduced anxiety was observed in wild-type littermates treated with anatabine in the elevated plus maze. In addition, social interaction and social memory deficits which are particularly pronounced in Tg PS1/APPswe were also mitigated by the anatabine treatment. Interestingly, the hippocampus has been shown recently to play an essential role in social memory [46] while lower levels of phosphorylated cAMP response element-binding protein (pCREB) in hippocampal neurons have been shown to reduce social recognition memory [47] highlighting the importance of normal hippocampal functions for social memory. Our observation that anatabine rescues social memory deficits in Tg PS1/APPswe may therefore suggest an improvement of hippocampal functions following treatment with anatabine in Tg PS1/APPswe mice. A recent study has also revealed that anatabine improves attention deficit in a rat model suggesting cognitive enhancing effects [48]. AD patients do present impaired social cognition and this abnormal decoding of social information has been linked with the conversion from prodromal AD to dementia [49] suggesting that the abnormal social interaction and memory observed in Tg PS1/APPswe is related to the AD pathophysiology. The prefrontal cortex and hippocampus have been shown previously to be involved in hyperactive behaviors [50] and mouse models of AD overexpressing Aβ commonly display hyperactivity [30]. Most AD patients have also been shown to display agitation and higher motor activity [51,52] suggesting that the observation of hyperactive behavior in mouse models of AD may also be relevant to the pathobiology of AD. We observed that Tg PS1/APPswe mice were effectively hyperactive in the elevated plus maze compared to wild-type littermates while anatabine significantly inhibited hyperactivity in Tg PS1/APPswe mice.

Anatabine displays a chemical structure relatively similar to nicotine and it is therefore expected to impact nicotinic acetylcholine receptors (nAChR). Anatabine has been shown to be a full agonist of α7 and α4β2 nAChR but is a more potent α7 nAChR agonist than nicotine [15,16]. Agonists of α7 nAChR are considered promising compounds for the treatment of AD and have been shown to lower Aβ deposition and to reverse long-term potentiation deficits induced by synaptotoxic Aβ oligomers [53–56]. nACh receptor agonists including nicotine have been shown to lower Aβ production *in vitro* [54] and Aβ deposition in the brain of transgenic mouse models of AD overexpressing Aβ [55,56]. Nicotine has also been shown to improve cognition in young Tg PS1/APPswe mice as well as in older mice with extensive Aβ deposition and to prevent synaptical impairments induced by Aβ oligomers [57]. Recent data have shown that a selective α7 nAChR agonist completely restored cognition in an aged triple transgenic mice model of AD (3x Tg-AD) with severe AD-like pathology [58] suggesting that stimulation of α7 nAChR may oppose cognitive deficits induced by both tau and β-amyloid pathologies.

Interestingly, a selective α7 nAChR agonist has been shown to improve rat social recognition memory and to reduce tau hyperphosphorylation [59] suggesting that nAChR agonists may also slow down tau pathology in AD. We have recently shown that anatabine reduces tau hyperphosphorylation and oligomerization using a pure model of tauopathy [21] further supporting this contention. BACE-1 elevation and reduced expression of nAChR α7 and β2 subunits are established hallmarks of AD and appear to be alleviated by nicotine administration in an Aβ infused rat model of AD [60,61]. Chronic transdermal delivery of nicotine has been shown to improve cognitive performances in patients with mild cognitive impairment [62]. In fact, tobacco smoking is associated with a reduction in Aβ deposition in the brain of normal elderly individuals [63]. In addition, reduced Aβ levels have been shown in the brain of smoking control and AD patients [64] further suggesting that stimulation of nAChR may also affect Aβ deposition in human. Nicotinic AChRs agonists could therefore represent promising compounds for the treatment of AD since they may have the ability to provide symptomatic relief and to slow down the pathology of AD.

Altogether, our data show that a chronic oral treatment with anatabine reduces β-amyloidosis and neuroinflammation and alleviates some behavioral impairments in Tg PS1/APPswe supporting further exploration of anatabine as a possible disease modifying agent for the treatment of AD.

## Supporting Information

S1 Fig Performances of wild-type and Tg PS1/APPswe receiving regular drinking water or anatabine in the radial arm water maze (RAWM). The data represents mean ± SEM. A) The graph represents the average number of errors made by Tg PS1/APPswe and wild-type mice receiving regular drinking water to find the hidden platform in RAWM. ANOVA showed a significant effect of genotype (P>0.001) for the number of the errors in the RAWM. Post hoc comparisons show that Tg PS1/APPswe mice receiving regular drinking water (placebo) made significantly more errors compared to wild-type littermates (P<0.001) showing reference memory impairment. Overall, wild-type mice made fewer errors (>2) than Tg PS1/APPswe mice (<3) in the RAWM suggesting better learning and cognitive performance by day 5. B) The graph represents the average number of errors made by wild-type mice receiving either regular drinking water (placebo) or anatabine at the dosage of 10 or 20 mg/Kg/Day dissolved in their drinking water. The performances of wild-type mice receiving anatabine at a dosage of 10 and 20 mg/Kg/Day were not significantly different from wild-type placebo mice (P>0.05). C) The graph represents the average number of errors made by Tg PS1/APPswe mice receiving either regular drinking water (placebo) or anatabine at the dosage of 10 or 20 mg/Kg/Day in their drinking water. Anatabine at either 10 mg/Kg/Day or 20 mg/Kg/Day did not significantly affect the number of errors elicited by Tg PS1/APPswe to find the hidden platform (P>0.05) in the RAWM suggesting that anatabine does not improve reference memory in Tg PS1/APPswe mice.
(TIF)

S2 Fig Distance travelled by Tg PS1/APPswe mice and their control wild-type littermates to find the hidden platform in the Morris water maze. The data represents mean ± SEM. A) The graph presents the distance travelled (in cm) by Tg PS1/APPswe placebo and control wild-type littermates across five days of testing (average of four trials per day). ANOVA showed a significant effect of genotype (P<0.001) and days of learning (P<0.001) for the distance travelled to find the platform. Control wild-type mice travelled less distance to find the hidden platform compared to Tg PS1/APPswe mice (P<0.001) showing that Tg PS1/APPswe mice elicit spatial working memory deficits in the Morris water maze. B) The graph presents the distance travelled by Tg PS1/APPswe mice receiving regular drinking water (placebo) or anatabine at a dosage of 10 or 20 mg/Kg/Day. No significant effect of the anatabine treatment (10 and 20mg/Kg/Day) was observed (P>0.05) in Tg PS1/APPswe mice on the average distance travelled by the mice to locate the hidden platform showing that anatabine does not improve spatial working memory in the Morris water maze.
(TIF)
