# Correction: Chronic Anatabine Treatment Reduces Alzheimer's Disease (AD)-Like Pathology and Improves Socio-Behavioral Deficits in a Transgenic Mouse Model of AD

**DOI:** 10.1371/journal.pone.0134776

**Published:** 2015-07-31

**Authors:** Megha Verma, David Beaulieu-Abdelahad, Ghania Ait-Ghezala, Rena Li, Fiona Crawford, Michael Mullan, Daniel Paris

The following information is missing from the Funding section: This study was also funded by the Roskamp Foundation.
