# Supplementary figures and images for: Chronic Anatabine Treatment Reduces Alzheimer’s Disease (AD)-Like Pathology and Improves Socio-Behavioral Deficits in a Transgenic Mouse Model of AD
Source: PLoS One. 2015 May 26;10(5):e0128224. doi: 10.1371/journal.pone.0128224 (PMC4444019; doi:10.1371/journal.pone.0128224)

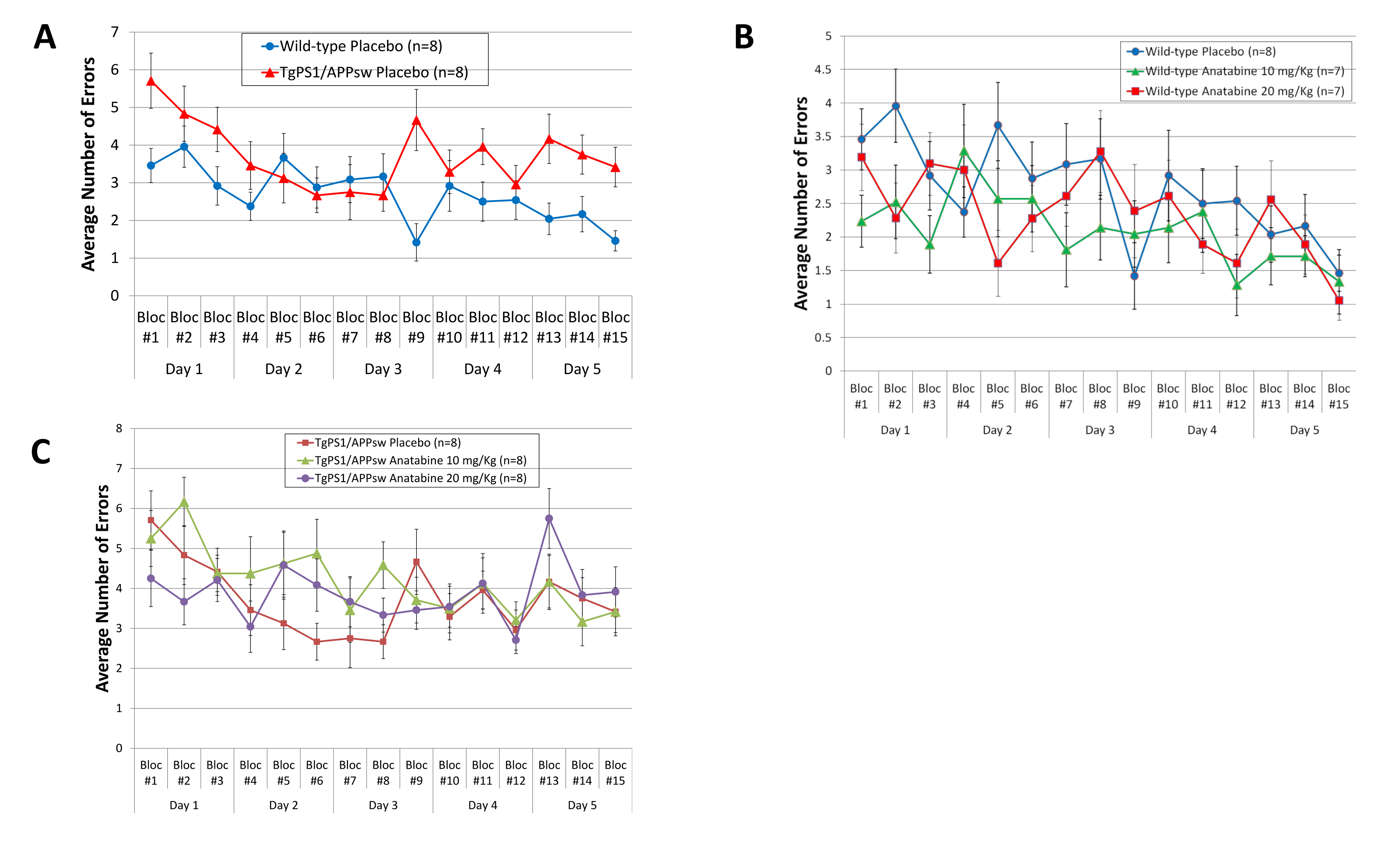

Supplement: S1 Fig — The data represents mean ± SEM. A) The graph represents the average number of errors made by Tg PS1/APPswe and wild-type mice receiving regular drinking water to find the hidden platform in RAWM. ANOVA showed a significant effect of genotype (P>0.001) for the number of the errors in the RAWM. Post hoc comparisons show that Tg PS1/APPswe mice receiving regular drinking water (placebo) made significantly more errors compared to wild-type littermates (P<0.001) showing reference memory impairment. Overall, wild-type mice made fewer errors (>2) than Tg PS1/APPswe mice (<3) in the RAWM suggesting better learning and cognitive performance by day 5. B) The graph represents the average number of errors made by wild-type mice receiving either regular drinking water (placebo) or anatabine at the dosage of 10 or 20 mg/Kg/Day dissolved in their drinking water. The performances of wild-type mice receiving anatabine at a dosage of 10 and 20 mg/Kg/Day were not significantly different from wild-type placebo mice (P>0.05). C) The graph represents the average number of errors made by Tg PS1/APPswe mice receiving either regular drinking water (placebo) or anatabine at the dosage of 10 or 20 mg/Kg/Day in their drinking water. Anatabine at either 10 mg/Kg/Day or 20 mg/Kg/Day did not significantly affect the number of errors elicited by Tg PS1/APPswe to find the hidden platform (P>0.05) in the RAWM suggesting that anatabine does not improve reference memory in Tg PS1/APPswe mice. (TIF) [file pone.0128224.s001.tif]

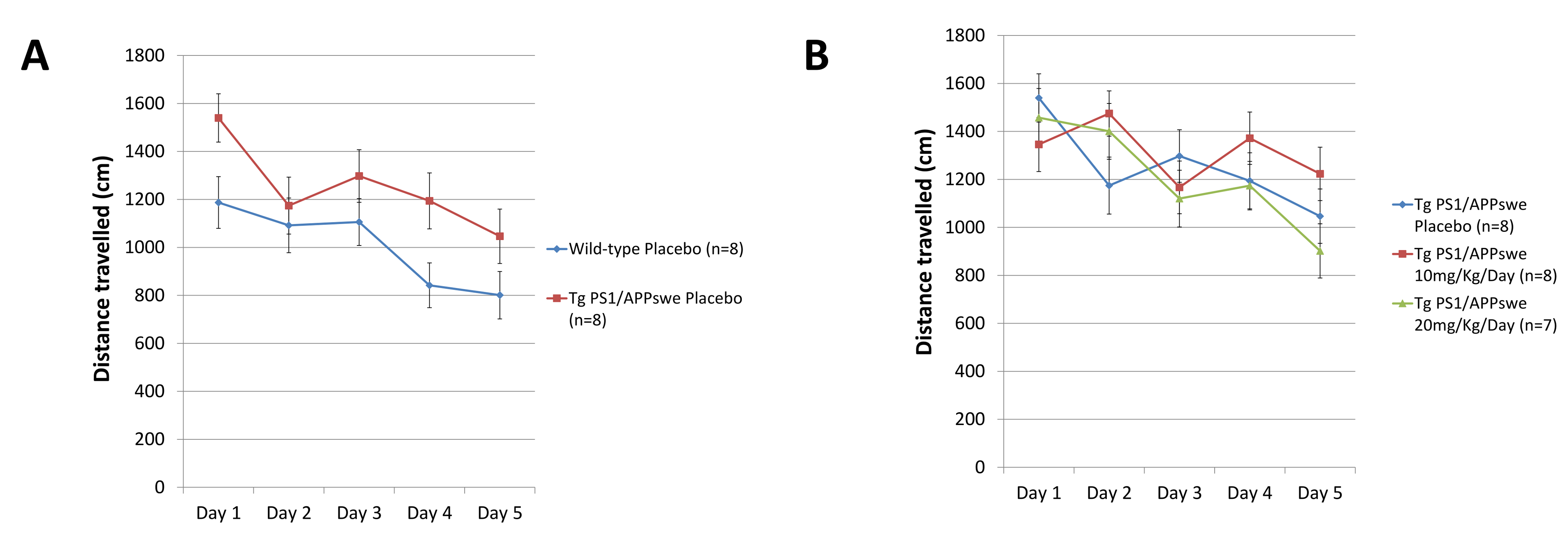

Supplement: S2 Fig — The data represents mean ± SEM. A) The graph presents the distance travelled (in cm) by Tg PS1/APPswe placebo and control wild-type littermates across five days of testing (average of four trials per day). ANOVA showed a significant effect of genotype (P<0.001) and days of learning (P<0.001) for the distance travelled to find the platform. Control wild-type mice travelled less distance to find the hidden platform compared to Tg PS1/APPswe mice (P<0.001) showing that Tg PS1/APPswe mice elicit spatial working memory deficits in the Morris water maze. B) The graph presents the distance travelled by Tg PS1/APPswe mice receiving regular drinking water (placebo) or anatabine at a dosage of 10 or 20 mg/Kg/Day. No significant effect of the anatabine treatment (10 and 20mg/Kg/Day) was observed (P>0.05) in Tg PS1/APPswe mice on the average distance travelled by the mice to locate the hidden platform showing that anatabine does not improve spatial working memory in the Morris water maze. (TIF) [file pone.0128224.s002.tif]
